# Stability of important antibodies for kidney disease: pre-analytic methodological considerations

**DOI:** 10.7717/peerj.5178

**Published:** 2018-07-09

**Authors:** Qiuxia Han, Songyan Li, Bo Fu, Dongwei Liu, Maoqing Wu, Xiaoli Yang, Guangyan Cai, Zhangsuo Liu, Xiangmei Chen, Hanyu Zhu

**Affiliations:** 1 Department of Nephrology, Chinese PLA General Hospital, Chinese PLA Institute of Nephrology, State Key Laboratory of Kidney Diseases, National Clinical Research Center for Kidney Diseases, Beijing Key Laboratory of Kidney Disease, Beijing, China; 2 Department of Nephrology of Zhenghou University, The First Affiliated Hospital of Zhengzhou University, Research Institute of Nephrology of Zhengzhou University, Key Laboratory of Precision Diagnosis and Treatment for Chronic Kidney Disease in Henan Province, Zhengzhou, China; 3 Department of General Surgery, Chinese PLA General Hospital, Beijing, China; 4 Center for Translational Science, Children’s National Medical Center, Washington DC, USA

**Keywords:** anti-PLA2R antibodies, anti-GBM antibodies, anti-MPO antibodies, anti-PR3 antibodies, Storage, Stability

## Abstract

**Background:**

The importance of circulating antibodies as biomarkers of kidney disease has recently been recognized. However, no study has systematically described the methodology of sample preparation and storage regarding antibodies as biomarkers of kidney disease. It remains unknown whether repetitive freeze-thaw cycles, physical disturbances, storage at different temperatures or for different periods of time, or haemolytic or turbid serum samples affect antibody measurements. The aim of this study was to investigate the stabilities of antibodies associated with kidney disease in serum samples under various relevant clinical and research conditions.

**Methods:**

We stored serum samples in the following different conditions: repetitive freeze-thaw cycles (1, 6 or 12 times), long-term storage (7 or 12 months at −80 °C), physical disturbance (1 or 8 h), and storage at 4 °C (1, 3 or 6 weeks) and room temperature (1 or 7 days). The stabilities of the anti-phospholipase A2 receptor (anti-PLA2R), anti-glomerular basement membrane, anti-myeloperoxidase and anti-proteinase 3 antibodies were evaluated with enzyme-linked immunosorbent assays (ELISA).

**Results:**

We found that repetitive freeze-thaw cycles did not have a significant effect on the stabilities of the abovementioned antibodies in clear serum samples. The ELISA readings of haemolytic and turbid serum samples tended to increase and decrease, respectively. Neither long-term storage at −80 °C nor physical disturbance had a significant effect on anti-PLA2R antibody stability in sealed serum samples. The concentrations of most of these antibodies increased in unsealed serum samples that were stored at 4 °C for more than 6 weeks or at room temperature for more than 7 days.

**Discussion:**

Our findings revealed that the abovementioned circulating antibodies that are used as biomarkers for kidney disease had stable physicochemical properties, structures and immunoreactivities such that they were not influenced by repetitive freeze-thaw cycles, physical disturbances or long-term storage at −80 °C. However, the ELISA readings tended to change for haemolytic, turbid and unsealed serum samples.

## Introduction

The potential importance of circulating antibodies as non-invasive diagnostic and predictive biomarkers has recently been recognized, especially for kidney disease. For example, the utility of M-type phospholipase A2 receptor (anti-PLA2R) antibodies for the diagnosis of idiopathic membranous nephropathy and the utility of anti-glomerular basement membrane (anti-GBM) antibodies for the diagnosis rapidly progressive glomerulonephritis and Goodpasture disease have been established. Additionally, the levels of anti-myeloperoxidase (anti-MPO) and anti-proteinase 3 (anti-PR3) antibodies are useful to diagnose antineutrophil cytoplasmic antibodies associated with systemic vasculitis and related kidney damage (Beck et al., 2009; McAdoo & Pusey, 2017; Yang et al., 2007). Many of these antibodies have been used as biomarkers for disease prognosis, diagnosis and even have been vital in guiding treatment strategies (Kanzaki et al., 2016; Li et al., 2016; Lin et al., 2016; Ohlsson et al., 2014; Savige et al., 1993; Watanabe et al., 2010; Weiner & Segelmark, 2016). Nonetheless, when antibodies are used as clinical biomarkers, attention must be paid to their stabilities given the various processing conditions of clinical samples.

In general, collected serum samples should be tested in a timely manner. However, when serum samples collected by grass-roots hospitals cannot perform special clinical detection methods, such as enzyme-linked immunosorbent assay (ELISA), the samples must be stored at a low temperature for often a long period of time before they are transported to an eligible test center. Therefore, serum samples may need be stored at 4 °C or room temperature for a short time or frozen for a long time. In addition, when detection of multiple indicators is required, samples need to be tested after repetitive freeze-thaw cycles. Moreover, serum samples are not always high quality. Some samples, such as haemolytic or turbid serum samples, have special characteristics. At times, serum is forcibly separated from uncoagulated blood to save time and quickly perform tests, which can easily lead to haemolysis within the sample. As another concern, the incidence of hyperlipidaemia has rapidly increased in recent years due to an increasing number of patients with dyslipidaemia caused by diabetes mellitus and familial hyperlipidaemia. Serum may exhibit varying degrees of turbidity at serum triglyceride concentrations above 3.0 mmol/L. Severe lipid opacity may seriously interfere with the detection of antibodies. Overall, the influences of these factors on ELISA, the most common detection method, is unclear.

Previous studies have suggested that confounding methodological factors should be ruled out (Chaigneau et al., 2007; Hodgkinson et al., 2017). To the best of our knowledge, no study has systematically described the methodology of sample preparation and storage with respect to important antibodies as blood biomarkers of kidney disease. Indeed, it remains unknown whether repetitive freeze-thaw cycles, physical disturbance, storage at different temperatures or for different periods of time, haemolytic or turbid serum samples affect antibody measurements and the reproducibility of results. Therefore, the aim of this study was to investigate the stability of antibody concentrations in serum under various research conditions, such as long-term storage at −80 °C, repetitive freeze-thaw cycles or physical disturbance, and clinical conditions, such as storage at 4 °C or room temperature.

## Materials and Methods

### Study population and serum sample collection

Serum samples were collected from Chinese PLA General Hospital patients after clinical tests. All selected patients (*n* = 454) were Chinese individuals, and most of the patients (*n* = 320) were antibody positive. Research related to humans complied with all relevant national regulations and institutional policies; the study was conducted in accordance with the tenets of the Helsinki Declaration and was approved by the Clinical Research Ethical Committee of Chinese PLA General Hospital. Informed consent was obtained from all individuals included in this study.

### Sample preparation and storage

Serum samples were obtained by collecting venous blood into Vacutainer serum separator tubes (Greiner Bio-One, Frickenhausen, Germany), and the samples were then centrifuged at 3,000 revolutions per minute (rpm) at 4 °C for 10 min in a centrifuge (Sigma-Aldrich, St. Louis, MO, USA) within 20 min. We collected clear (neither turbid nor haemolytic) serum, turbid serum and haemolytic serum. In order to mimic haemolysis, some venous blood samples were shaken for 5 min using a Vortex (Vortex-Genie; Scientific Industries, Bohemia, NY, USA). The concentrations of anti-PLA2R antibodies, anti-GBM antibodies, anti-MPO antibodies and anti-PR3 antibodies were measured using ELISA kits and a EUROIMMUN Analyzer I machine (Euroimmun, Lübeck, Germany). All four of these antibodies were positive for ELISA readings greater than 20 RU/mL. The linear detection range of the ELISA method for anti-PLA2R antibodies was 2–1,500 RU/mL, and the linear detection ranges for anti-GBM antibodies, anti-MPO antibodies and anti-PR3 antibodies were 2–200 RU/mL. Considering the measurement accuracy of detection, serum samples with antibody concentrations beyond the linear range were excluded from our study. The numbers of individuals and the raw data for each experiment are presented in the Supplementary Materials.

### Experimental procedures

#### Repetitive freeze-thaw cycles

We assessed anti-PLA2R antibodies in clear serum samples (*n* = 46), turbid serum samples (*n* = 20) and haemolytic serum samples (*n* = 20). The anti-GBM antibodies (*n* = 20), anti-MPO antibodies (*n* = 20) and anti-PR3 antibodies (*n* = 20) were evaluated in clear serum samples. Each serum sample was aliquoted into four 1.5 mL Eppendorf tubes (Axygen, Union City, CA, USA) that were then sealed with Parafilm (Pechiney, Chicago, IL, USA). One aliquot was frozen until it was thawed and analyzed, and the other aliquots were repeatedly frozen and thawed (frozen at −80 °C for at least 3 h to ensure complete freezing and thawed at room temperature for approximately 1 h to ensure to complete thawing) for 6 or 12 cycles prior to analysis.

#### Long-term storage at −80 °C

We collected serum samples from 22 patients, and measurements of freshly centrifuged samples were used as the controls for each of the cases. Then, we divided each of the samples into two 1.5 mL Eppendorf tubes. One aliquot was closed and sealed with Parafilm, and the other was closed but left unsealed. Both aliquots were stored at −80 °C for 7 months, after which testing was performed for anti-PLA2R antibodies. Serum samples from 21 other patients were collected and processed as described above and then stored at −80 °C for 12 months (closed but unsealed) prior to testing for anti-PLA2R antibodies.

#### Influence of physical and mechanical disturbances

We evaluated anti-PLA2R antibodies in clear serum samples from 21 patients, and freshly centrifuged sample measurements served as controls for each of the cases. Then, each sample was aliquoted into two 1.5 mL Eppendorf tubes and sealed with Parafilm. For each sample, one aliquot was stored at room temperature (19–21 °C), and the other sample was placed on an orbital shaker (Qilinbeier, Haimen, China) at 110 rpm at room temperature for 2 or 8 h of shaking prior to testing for anti-PLA2R antibodies.

#### Storage at 4 °C

Clear serum (*n* = 21), turbid serum (*n* = 20) and haemolytic serum (*n* = 20) samples were used to test for anti-PLA2R antibodies. Clear serum samples were used to test for anti-GBM (*n* = 21), anti-MPO (*n* = 20) and anti-PR3 (*n* = 20) antibodies. Samples in Vacutainer serum separator tubes were stored in parallel at 4 °C for 1, 3 or 6 weeks prior to analysis.

#### Storage at room temperature

We tested for anti-PLA2R antibodies in clear serum (*n* = 20), turbid serum (*n* = 20) and haemolytic serum (*n* = 21) samples, and we tested for anti-GBM (*n* = 21), anti-MPO (*n* = 20) and anti-PR3 (*n* = 20) antibodies in clear serum samples. Samples in Vacutainer serum separator tubes were stored in parallel at room temperature (19–21 °C) for 1 or 7 days prior to analysis.

### Statistical analysis

Normally distributed data were compared using the paired two-tailed *t*-test or repeated measures analysis of variance. Nonparametric variables were compared using the Kruskal–Wallis test. Statistical analyses were performed using SPSS statistical software, Version 19.0 (SPSS Inc., Chicago, IL, USA), and a *p* value (two-sided test) less than 0.05 was considered statistically significant.

## Results

### Antibody stability after repetitive freeze-thaw cycles

**Figure 1:**
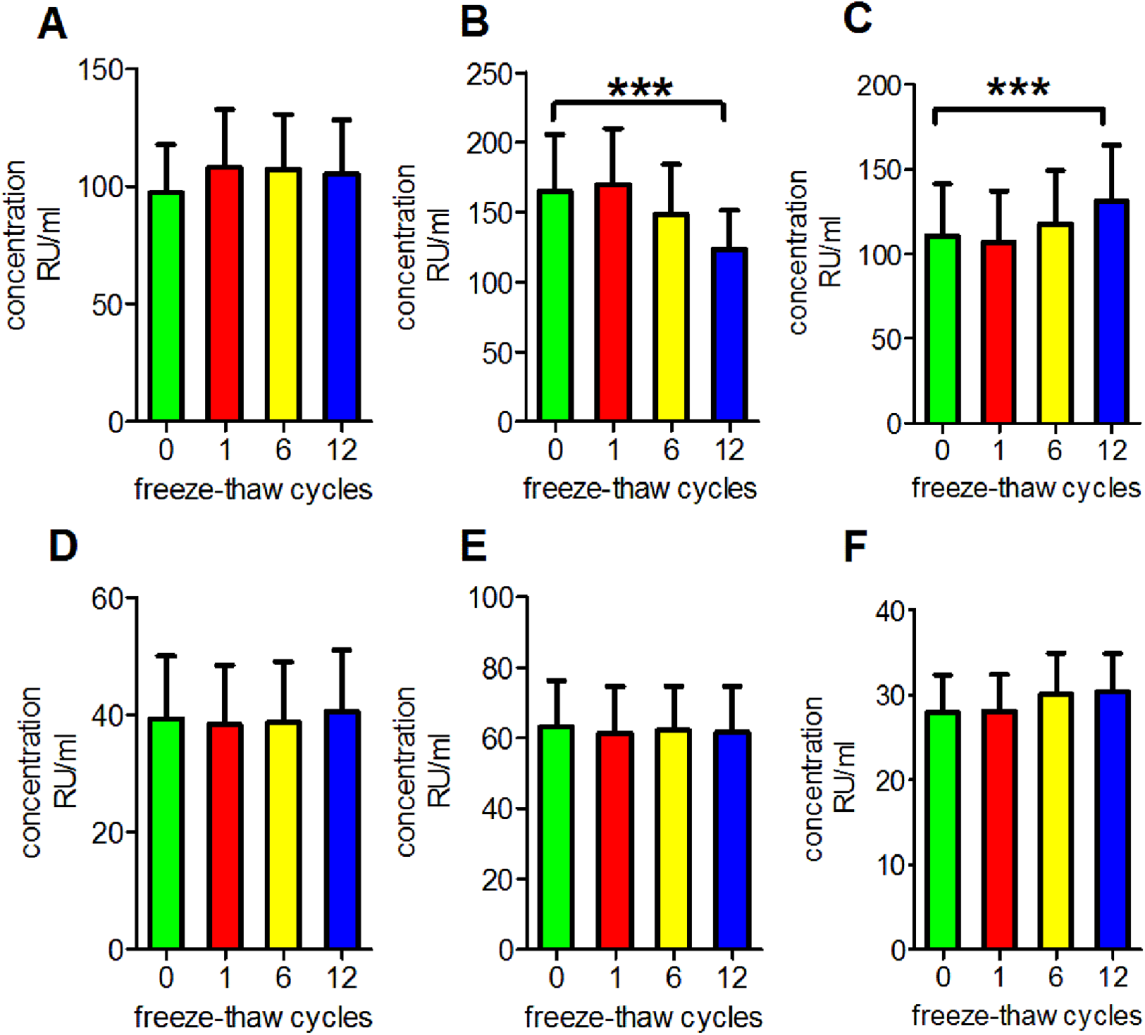
Influence of repetitive freeze-thaw cycles. (A) The reading of anti-PLA2R antibodies in clear serum samples. (B) The reading of anti-PLA2R antibodies in turbid serum samples. (C) The reading of anti-PLA2R antibodies in haemolytic serum samples. (D) The reading of anti-GBM antibodies in clear serum samples. (E) The reading of anti-MPO antibodies in clear serum samples. (F) The reading of anti-PR3 antibodies in clear serum samples. ***p < 0.001.

We performed a series of experiments to determine the reproducibility of the results under routine laboratory conditions and to evaluate the stability of antibodies in serum subjected to repetitive freeze-thaw cycles. We observed that repetitive freezing and thawing had no significant effect on the measurements of anti-PLA2R antibodies, anti-GBM antibodies, anti-MPO antibodies or anti-PR3 antibodies in clear serum samples (*p* = 0.9993, 0.9513, 0.9909 and 0.9553, respectively). However, anti-PLA2R antibodies measurements were decreased in turbid serum but were increased in haemolytic serum (*p* < 0.001 for both; Fig. 1).

### Antibody stability during long-term storage at −80 °C

**Figure 2:**
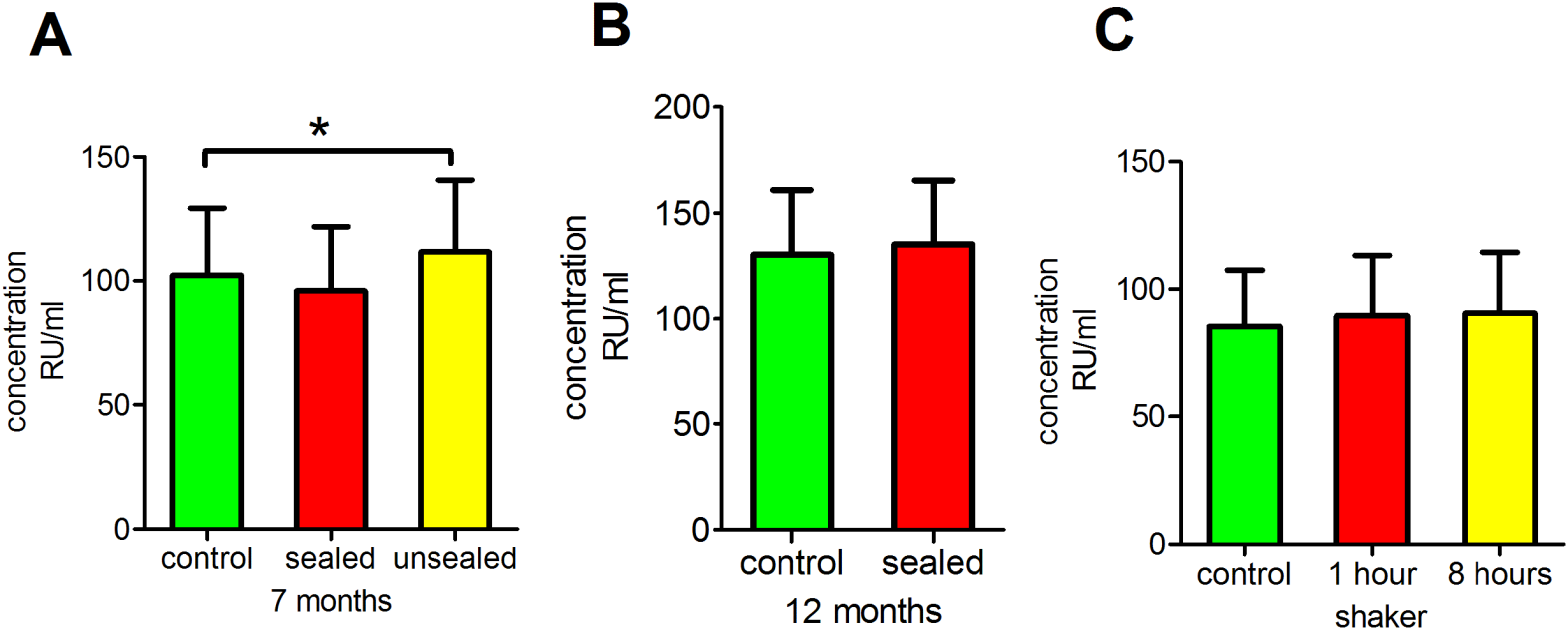
Influence of long-term storage at −80 °C and physical and mechanical disturbances. (A) Clear serum samples were stored at −80 °C for 7 months prior to testing anti-PLA2R antibodies. (B) Clear serum samples were stored at −80 °C for 12 months prior to testing anti-PLA2R antibodies. (C) Clear serum samples were placed on a shaker at 110 rpm at room temperature for 1 or 8 h of shaking prior to testing anti-PLA2R antibodies. *p < 0.05.

Next, we investigated whether long-term storage at −80 °C had any effect on anti-PLA2R antibody readings. This analysis revealed that the storage of sealed serum at −80 °C for 7 or 12 months had no significant effect on anti-PLA2R antibody concentrations (*p* = 0.9066 and 0.7820, respectively), whereas the storage of unsealed serum at −80 °C for 7 months did have a significant effect (*p* < 0.05; Figs. 2A and 2B).

### Antibody stability after physical and mechanical disturbance

We placed serum samples on a shaker for 2 or 8 h to evaluate the effect of physical disturbances. The results revealed that this physical disturbance had no significant effect on the anti-PLA2R antibody measurements (*p* = 0.9341; Fig. 2C).

### Incubation of serum at 4 °C for extended periods of time

**Figure 3:**
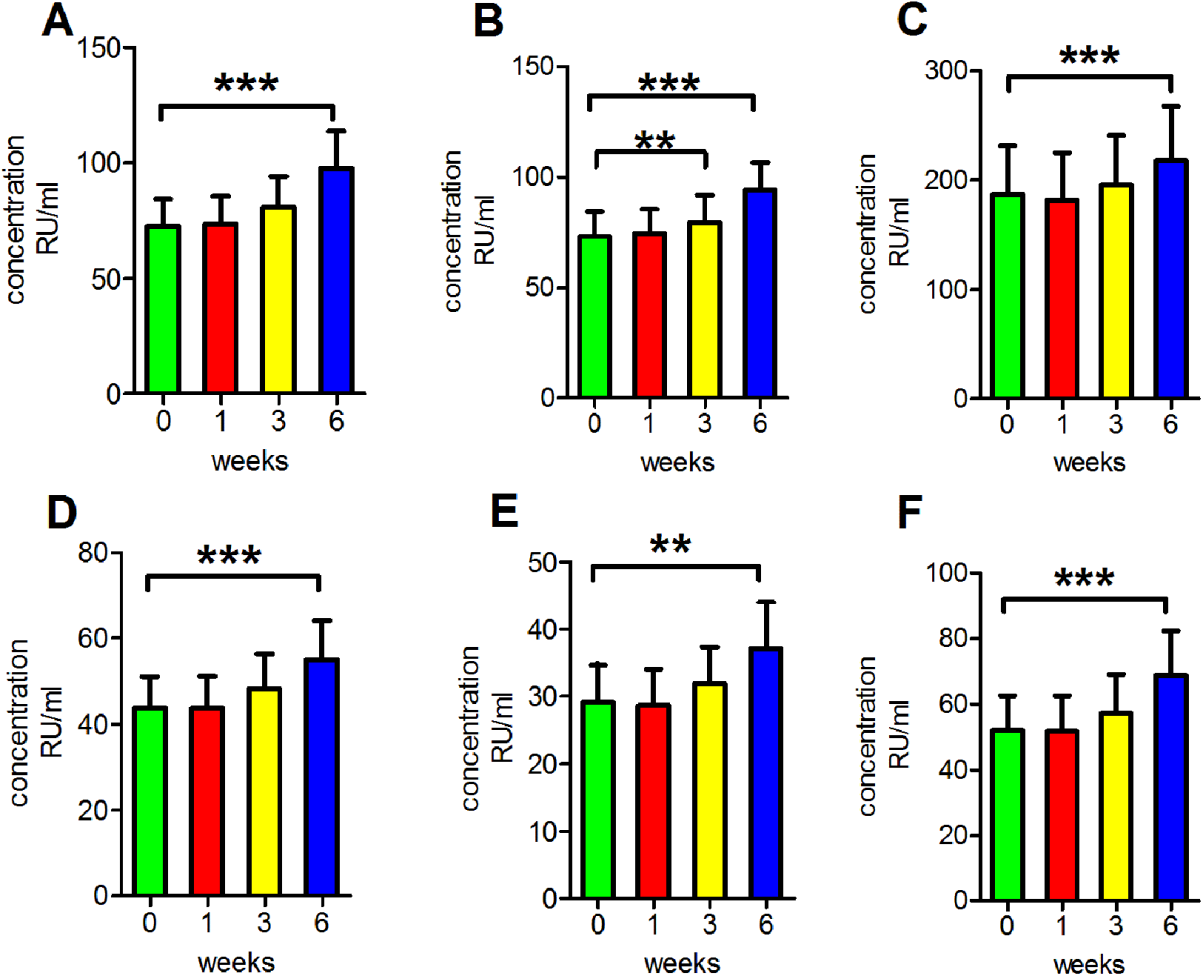
Influence of storage at −4 °C. (A) The reading of anti-PLA2R antibodies in clear serum samples. (B) The reading of anti-PLA2R antibodies in turbid serum samples. (C) The reading of anti-PLA2R antibodies in haemolytic serum samples. (D) The reading of anti-GBM antibodies in clear serum samples. (E) The reading of anti-MPO antibodies in clear serum samples. (F) The reading of anti-PR3 antibodies in clear serum samples. **p < 0.01, ***p < 0.001.

We then performed another series of experiments to determine the reproducibility of results under routine clinical conditions. The concentrations of antibodies were detected after incubation at 4 °C for various durations. This process was performed to mimic clinical serum sample handling when refrigeration is available. We found that the readings of anti-PLA2R antibodies in clear serum samples, turbid serum samples and haemolytic serum samples and the readings of anti-GBM antibodies, anti-MPO antibodies and anti-PR3 antibodies in clear serum samples all increased following incubation at 4 °C for 6 weeks, and the differences were significant (*p* < 0.001 for anti-PLA2R antibodies in clear serum samples, turbid serum samples and haemolytic serum samples and anti-GBM antibodies and anti-PR3 antibodies in clear serum samples; *p* < 0.01 for anti-MPO antibodies in clear serum samples). Additionally, the anti-PLA2R antibody readings in haemolytic serum were significantly increased following incubation at 4 °C for 3 weeks (*p* < 0.01; Fig. 3).

### Incubation of serum at room temperature for extended periods of time

**Figure 4:**
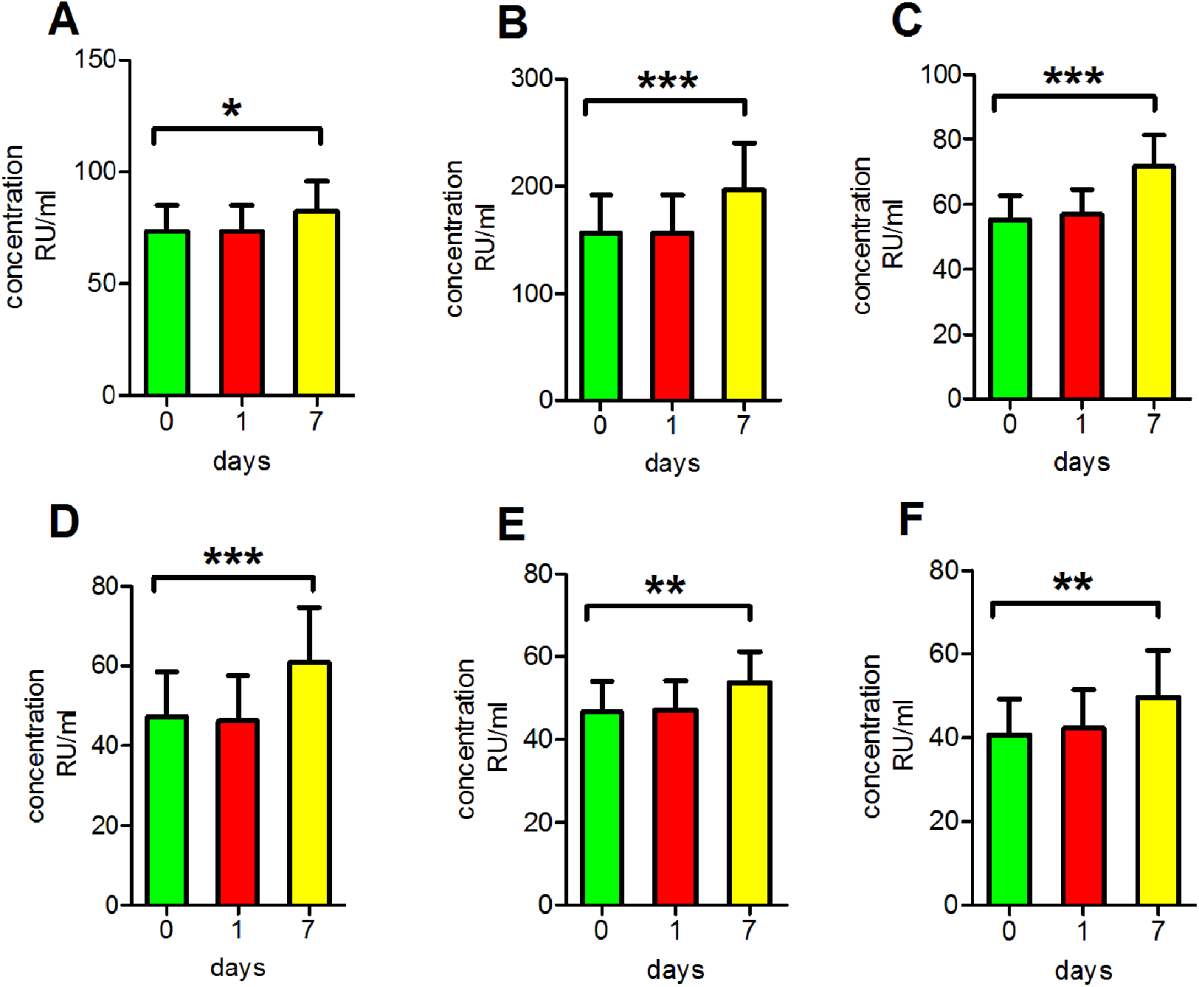
Influence of storage at room temperature. (A) The reading of anti-PLA2R antibodies in clear serum samples. (B) The reading of anti-PLA2R antibodies in turbid serum samples. (C) The reading of anti-PLA2R antibodies in haemolytic serum samples. (D) The reading of anti-GBM antibodies in clear serum samples. (E) The reading of anti-MPO antibodies in clear serum samples. (F) The reading of anti-PR3 antibodies in clear serum samples. *p < 0.05, **p < 0.01, ***p < 0.001.

To mimic clinical serum sample handling when refrigeration is unavailable, antibody concentrations after incubation at room temperature for various periods of time were assessed. We found that the readings of anti-PLA2R antibodies in clear serum samples, turbid serum samples and haemolytic serum samples as well as those of anti-GBM antibodies, anti-MPO antibodies and anti-PR3 antibodies in clear serum samples all increased significantly following incubation at room temperature for 7 days (*p* < 0.05 for anti-PLA2R antibodies in clear serum samples, *p* < 0.01 for anti-MPO antibodies and anti-PR3 antibodies in clear serum samples, *p* < 0.001 for anti-PLA2R antibodies in turbid serum samples and haemolytic serum samples and anti-GBM antibodies in clear serum samples; Fig. 4).

## Discussion

Inaccurate results of the laboratory tests can lead to misdiagnosis of diseases and illness severities that affect the medical decision-making of doctors. Our study highlights several variables that may affect measurements of antibodies in serum samples. To the best of our knowledge, this study is the first to provide detailed methodological information regarding the importance of antibody tests, which have been presumed to generate reliable and representative data for use in routine clinical applications and the diagnoses of various types of kidney disease.

The stabilities of antibodies we considered were based on ELISA readings and involved changes related to structure, immunoreactivity and concentration. When an antibody concentration is extremely low, changes that occur during extreme storage conditions are very small. For this reason, most of the patients in our study were antibody positive and exhibited high concentrations. Additionally, our results are reliable due to the use of automatic machines with extremely high accuracies.

### Antibody stability after repetitive freeze-thaw cycles

When the detection of several indicators is required, samples may need to be repeatedly frozen and thawed due to a limited number of aliquots in the biobanks. Consequently, denaturation, aggregation and functional loss of circulating proteins might occur (Chaigneau et al., 2007). Nonetheless, many proteins are stable after repetitive freeze-thaw cycles (Jarius & Wildemann, 2011; Lee, Kim & Shin, 2015). We observed that repetitive freeze-thaw cycles did not significantly affect the detections of anti-PLA2R, anti-GBM, anti-MPO or anti-PR3 antibodies in clear serum. Our results indicated that the structures and immunoreactivities of these antibodies are very stable, and repetitive freeze-thaw cycles did not influence their detection. These antibodies can thus be used as stable biomarkers for diagnosis regardless of repeated freezing and thawing.

Although anti-PLA2R antibodies in turbid serum exhibited a significant decrease in concentration, those in haemolytic serum exhibited the opposite tendency. The high triglyceride concentrations of serum samples increased the numbers of chylomicrons and increased the viscosity. Consequently, the molecular motion slowed, which reduced the probability of antigen and antibody binding. Haemolysis is caused by the rupture of erythrocytes, which results in the release of some components into the serum, including haemoglobin, peroxidase and ferroheme. The release of these components might have elicited a complicated influence on the serological test results. On the one hand, haemoglobin and peroxidase non-specifically adsorb to the reaction wells of the plates used in ELISA experiments, which increases the difficulty of the washing step. On the other hand, ferroheme from erythrocytes might cause abnormal chromogenic reactions. The above factors may increase the optical density, which would cause inconsistent results, and repetitive freeze-thaw cycles may exacerbate these effects (Frank et al., 1978; Sonntag, 1986). Therefore, samples should be collected and handled using standard operating procedures to prevent haemolysis, and repetitive freeze-thaw cycles should be avoided when using turbid serum samples.

### Antibody stability during long-term storage at −80 °C

Previous studies have demonstrated that many types of antibodies are stable for long times when they are stored sealed (Gomez-Morales et al., 2015; Michaut et al., 2014). We performed another series of experiments to determine the reproducibility of results under routine laboratory conditions and to evaluate the concentrations of antibodies in serum samples after long-term storage at −80 °C. Our findings were concordant with other publications and revealed that the storage of sealed serum at −80 °C for 7 or 12 months did not have a significant effect on the concentration of anti-PLA2R antibodies, whereas the storage of unsealed serum at −80 °C for 7 months did have a significant effect. These findings indicate that when serum samples are not sealed, long-term storage may cause evaporation, which increases antibody concentrations and leads to inconsistent results. Therefore, it is important to keep in mind that samples should be sealed if they need to be stored at −80 °C for long periods of time.

### Antibody stability during physical and mechanical disturbance

To evaluate the effect of physical disturbances, serum samples were placed on a shaker for 2 or 8 h to mimic the transport of serum samples from one center to another. The results suggested that physical disturbances had no significant effect on the detection of anti-PLA2R antibodies. This result indicates that the structures and immunoreactivities of antibodies are very stable and not influenced by physical disturbances. Therefore, these antibodies may be used as stable biomarkers.

### Incubation of serum at 4 °C for extended periods of time

Previous studies have demonstrated that antibody concentrations in the serum are stable at 4 °C for short times (Hodgkinson et al., 2017; Rodriguez-Capote et al., 2016). Our study results agree with those of previous reports. We found that the measurements of anti-PLA2R antibodies in clear serum, turbid serum and haemolytic serum samples and those of anti-GBM, anti-MPO and anti-PR3 antibodies in clear serum samples were all increased following incubated at 4 °C for 6 weeks. However, the readings of anti-PLA2R antibodies in haemolytic serum were significantly increased in just the third week of incubation at 4 °C. These results indicated that the storage of serum at 4 °C under clinical conditions for extended periods of time (within 3 weeks) may not severely affect serum antibody detection. However, when serum is stored for 6 weeks or longer, antibody concentration exhibits an increasing trend. A possible reason may be that a very long period of storage, such as 6 weeks or longer, could cause evaporation, which would then increase the antibody concentration and lead to inconsistent results. Our serum samples were stored in Vacutainers that were not completely sealed. In view of these results, it is important to keep in mind that samples should not be stored at 4 °C for extended periods; if the storage period is 6 weeks or longer, the samples should be frozen for storage.

### Incubation of serum at room temperature for extended periods of time

We found that most serum samples became feculent and milky following storage at room temperature for 7 days or longer. In addition, measurements of anti-PLA2R antibodies in clear serum, turbid serum and haemolytic serum samples as well as measurements of anti-GBM, anti-MPO and anti-PR3 antibodies in clear serum samples all significantly increased following incubation at room temperature for 7 days. A possible explanation is that the serum samples were contaminated with bacteria that produced horseradish and peroxidase, which may have caused non-specific color changes and thus increased detection and led to inconsistent results (Kok & Wild, 1960; Watanabe et al., 1978). In view of these results, an important consideration is that samples should not be stored at room temperature for extended periods because they can easily become contaminated by bacteria. If the storage period is long, samples should be refrigerated or frozen for storage.

## Conclusion

These findings are particularly important for epidemiologic studies recruiting participants from remote, grass-roots hospitals where sample exposure to pre-analytic conditions can vary considerably. As detection of these antibodies is significantly altered by storage conditions, the conditions need to be standardized for epidemiological and clinical studies, and comparisons between studies may not be reliable. Our study suggested that non-standard processes should be avoided to overcome the challenges related to antibody stability and thus enable the use of antibodies as non-invasive diagnostic biomarkers that provide valid and comparable results. For example, when serum samples are turbid or haemolytic, repetitive freeze-thaw cycles should be avoided. If serum samples need to be stored at −80 °C for several months, they should be sealed well. If serum samples need to be stored at 4 °C, storage should not exceed 3 weeks. If refrigeration is unavailable and serum samples need to be stored at room temperature, storage should not exceed 7 days.

## Supplemental Information

10.7717/peerj.5178/supp-1 Supplemental Information 1 Raw data.

## Additional Information and Declarations

### Data Availability

The following information was supplied regarding data availability: The raw data obtained during experiments are provided in the Supplemental Files.
