# Supplementary material for: Stability of important antibodies for kidney disease: pre-analytic methodological considerations
Source: PeerJ. 2018 Jul 9;6:e5178. doi: 10.7717/peerj.5178 (PMC6042478; doi:10.7717/peerj.5178)
Supplement: Supplemental Information 1 [file peerj-06-5178-s001.docx]

**Table 1 .Repetitive freeze-thaw cycles:** **the reading of anti-PLA2R antibodies in clear serum samples.**

| Patients | 0 cycle | 1 cycle | 6 cycles | 12 cycles |
| --- | --- | --- | --- | --- |
| 1 | 27.92 | 25.69 | 37.51 | 26.61 |
| 2 | 3.1 | 3.36 | 3.84 | 4.09 |
| 3 | 655.61 | 785.21 | 729.72 | 635.28 |
| 4 | 127.07 | 153.77 | 176.52 | 189.79 |
| 5 | 93.35 | 99.5 | 105.97 | 89.48 |
| 6 | 10.88 | 10.1 | 11.85 | 9.22 |
| 7 | 138.67 | 224.68 | 253.71 | 137.83 |
| 8 | 104.27 | 119.53 | 155.54 | 88.37 |
| 9 | 84.32 | 82 | 118 | 70.06 |
| 10 | 36.31 | 27.58 | 33.61 | 34.9 |
| 11 | 327.38 | 332.22 | 479.28 | 377.58 |
| 12 | 539.17 | 744.58 | 598.62 | 726.44 |
| 13 | 103.89 | 152.35 | 133.6 | 197.46 |
| 14 | 6.99 | 7.04 | 6.59 | 6.69 |
| 15 | 265.45 | 348.88 | 267.35 | 390.84 |
| 16 | 58.9 | 57.67 | 52.73 | 54.32 |
| 17 | 32.84 | 27.58 | 23.76 | 39.52 |
| 18 | 22.61 | 22.01 | 28.57 | 27.47 |
| 19 | 19.88 | 17.78 | 17.88 | 24.45 |
| 20 | 44.19 | 37.38 | 34.94 | 38.05 |
| 21 | 16.79 | 15.15 | 15.05 | 13.19 |
| 22 | 11.11 | 10.24 | 11.97 | 10.44 |
| 23 | 74.55 | 72.09 | 66.39 | 89.53 |
| 24 | 9.57 | 8.77 | 10.33 | 10.79 |
| 25 | 6.38 | 5.07 | 4.71 | 6.91 |
| 26 | 18.24 | 22.42 | 18.28 | 25.22 |
| 27 | 83.22 | 72.72 | 109.33 | 105.27 |
| 28 | 36.23 | 45.43 | 39.28 | 62.4 |
| 29 | 49 | 53.31 | 51.52 | 54.22 |
| 30 | 29.14 | 33.61 | 33.56 | 34.25 |
| 31 | 200.48 | 221.16 | 161.15 | 146.12 |
| 32 | 29.88 | 42.52 | 26.19 | 29.32 |
| 33 | 126.53 | 146.84 | 134.34 | 171.98 |
| 34 | 153.45 | 189.5 | 133.36 | 136.58 |
| 35 | 5.68 | 6.09 | 5.07 | 6.14 |
| 36 | 66.29 | 96 | 58.98 | 70.21 |
| 37 | 391.86 | 312.85 | 370.94 | 354.69 |
| 38 | 16.83 | 19.29 | 17.48 | 14.1 |
| 39 | 48.64 | 36.68 | 48.59 | 39.04 |
| 40 | 254.73 | 156.55 | 225.57 | 169.51 |
| 41 | 54.97 | 45.09 | 48.31 | 36.96 |
| 42 | 20.48 | 20.74 | 20.47 | 21.02 |
| 43 | 12.32 | 8.37 | 11.36 | 9.04 |
| 44 | 19.21 | 14.63 | 17.83 | 15.85 |
| 45 | 25.58 | 19.11 | 21.57 | 25.35 |
| 46 | 12.45 | 14.74 | 13.52 | 14.4 |

**Table 2 .Repetitive freeze-thaw cycles:** **The reading of anti-PLA2R antibodies in turbid serum samples.**

| Patients | 0 cycle | 1 cycle | 6 cycles | 12 cycles |
| --- | --- | --- | --- | --- |
| 1 | 233.79 | 249.58 | 219.66 | 154.78 |
| 2 | 280.81 | 278.6 | 240.47 | 189.5 |
| 3 | 310.6 | 347.99 | 306.41 | 253.37 |
| 4 | 18 | 19.73 | 17.77 | 19.12 |
| 5 | 11.4 | 12.78 | 10.1 | 10.46 |
| 6 | 21.57 | 23.03 | 23.39 | 18.63 |
| 7 | 673.91 | 615.72 | 583.98 | 429.65 |
| 8 | 494.89 | 507.89 | 389.64 | 312.19 |
| 9 | 25.39 | 27.29 | 23.15 | 24.44 |
| 10 | 97.99 | 106.17 | 99.59 | 69.78 |
| 11 | 16.5 | 11.74 | 10.53 | 8.3 |
| 12 | 43.89 | 39.58 | 40.62 | 39.48 |
| 13 | 111.95 | 130.5 | 99.47 | 89.42 |
| 14 | 328.57 | 386.3 | 350.47 | 297.49 |
| 15 | 59.48 | 68.94 | 42.86 | 39.97 |
| 16 | 11.53 | 15.37 | 9.47 | 14.42 |
| 17 | 32.77 | 32.95 | 35.86 | 33.73 |
| 18 | 119.47 | 122.86 | 108.49 | 95.38 |
| 19 | 164.9 | 145.8 | 120.58 | 125.36 |
| 20 | 246.72 | 255.84 | 238.04 | 248.41 |

**Table 3 .Repetitive freeze-thaw cycles:** **The reading of anti-PLA2R antibodies in haemolytic serum samples..**

| Patients | 0 cycle | 1 cycle | 6 cycles | 12 cycles |
| --- | --- | --- | --- | --- |
| 1 | 43.96 | 42.49 | 60.78 | 81.38 |
| 2 | 27.53 | 40.43 | 39.92 | 48.58 |
| 3 | 8.1 | 4.98 | 4.82 | 4.52 |
| 4 | 20.92 | 22.94 | 21.69 | 28.49 |
| 5 | 14.17 | 14.98 | 12.81 | 24.07 |
| 6 | 115.84 | 120.59 | 135.26 | 142.74 |
| 7 | 163.96 | 168.39 | 175.48 | 192.73 |
| 8 | 20.63 | 17.63 | 21.94 | 29 |
| 9 | 33.09 | 37.26 | 59.3 | 58.42 |
| 10 | 18.53 | 20.45 | 28.96 | 35.71 |
| 11 | 52.66 | 49.11 | 64.94 | 83.66 |
| 12 | 472.93 | 480.62 | 477.34 | 496.3 |
| 13 | 288.49 | 264.96 | 274.36 | 310.48 |
| 14 | 53.9 | 49.72 | 64.3 | 72.84 |
| 15 | 311.96 | 301.42 | 349.84 | 375.39 |
| 16 | 58.93 | 51.82 | 55.25 | 71.94 |
| 17 | 69.07 | 17.77 | 37.3 | 40.59 |
| 18 | 47.77 | 53.69 | 66.66 | 93.03 |
| 19 | 16.62 | 18.28 | 23.73 | 28.92 |
| 20 | 372.88 | 359.31 | 385.39 | 410.42 |

**Table 4 .Repetitive freeze-thaw cycles: The reading of anti-GBM antibodies in clear serum samples.**

| Patients | 0 cycle | 1 cycle | 6 cycles | 12 cycles |
| --- | --- | --- | --- | --- |
| 1 | 17.38 | 18.29 | 18.45 | 21.36 |
| 2 | 5.29 | 4.61 | 5.1 | 6.4 |
| 3 | 15.79 | 13.89 | 16.74 | 24.65 |
| 4 | 17.38 | 13 | 12.02 | 16.9 |
| 5 | 18.27 | 17.73 | 17.72 | 15.44 |
| 6 | 25.31 | 34.53 | 32.14 | 31.92 |
| 7 | 54.7 | 56.77 | 56.43 | 60.18 |
| 8 | 8.09 | 6.72 | 7.95 | 9.25 |
| 9 | 22.54 | 19.84 | 20 | 22.04 |
| 10 | 107.01 | 98.31 | 99.33 | 109.66 |
| 11 | 6.77 | 5.5 | 5.75 | 6.4 |
| 12 | 44.01 | 36.46 | 41.68 | 43.83 |
| 13 | 102.12 | 113.29 | 112.72 | 107.73 |
| 14 | 50.54 | 50.53 | 48.37 | 46.67 |
| 15 | 57.69 | 52.91 | 50.53 | 49.96 |
| 16 | 11 | 14.95 | 14.87 | 15.52 |
| 17 | 11.14 | 15.03 | 13.89 | 16.99 |
| 18 | 197.54 | 182.3 | 186.72 | 191.95 |
| 19 | 6.01 | 6.32 | 5.99 | 7.05 |
| 20 | 7.31 | 5.1 | 7.86 | 8.03 |

**Table 5 .Repetitive freeze-thaw cycles:** **The reading of anti-MPO antibodies in clear serum samples.**

| Patients | 0 cycle | 1 cycle | 6 cycles | 12 cycles |
| --- | --- | --- | --- | --- |
| 1 | 36.69 | 35.94 | 41.72 | 36.07 |
| 2 | 11.98 | 11.74 | 11.47 | 11.95 |
| 3 | 93.2 | 95.19 | 95.31 | 96.95 |
| 4 | 190.31 | 198.62 | 180.38 | 195.79 |
| 5 | 19.46 | 20.5 | 15.53 | 19.42 |
| 6 | 87.03 | 84.9 | 88.28 | 80.63 |
| 7 | 56.17 | 44.98 | 45.48 | 41.09 |
| 8 | 7.14 | 6.4 | 7.28 | 6.33 |
| 9 | 60.79 | 46.61 | 63.18 | 56.15 |
| 10 | 12.96 | 12.15 | 13.84 | 12.88 |
| 11 | 194.1 | 183.43 | 191.84 | 194 |
| 12 | 166.11 | 166.86 | 155.69 | 156.69 |
| 13 | 41.46 | 46.11 | 45.86 | 43.93 |
| 14 | 96.19 | 96.57 | 90.04 | 90.57 |
| 15 | 52.26 | 43.72 | 54.27 | 53.39 |
| 16 | 12.69 | 18.85 | 18.49 | 16.93 |
| 17 | 14.72 | 11.41 | 12.89 | 14.25 |
| 18 | 53.07 | 54.39 | 50.25 | 58.91 |
| 19 | 9.3 | 9.84 | 10.32 | 8.97 |
| 20 | 46.83 | 39.72 | 51.49 | 36.2 |

**Table 6 .Repetitive freeze-thaw cycles: the reading of anti-PR3 antibodies in clear serum samples.**

| Patients | 0 cycle | 1 cycle | 6 cycles | 12 cycles |
| --- | --- | --- | --- | --- |
| 1 | 11.3 | 13.54 | 9.22 | 12.98 |
| 2 | 54.27 | 49.73 | 59.32 | 51.53 |
| 3 | 53.14 | 59.22 | 68.32 | 55.3 |
| 4 | 65.21 | 58.32 | 69.24 | 55.73 |
| 5 | 2.11 | 4.07 | 5.64 | 3.88 |
| 6 | 27.59 | 25.74 | 35.32 | 31.24 |
| 7 | 35.64 | 28.44 | 38.75 | 32.51 |
| 8 | 8.54 | 6.12 | 9.31 | 10.33 |
| 9 | 42.87 | 48.42 | 39.53 | 52.47 |
| 10 | 3.09 | 5.32 | 6.74 | 4.65 |
| 11 | 18.43 | 21.41 | 16.38 | 17.67 |
| 12 | 15.8 | 19.42 | 21.54 | 17.65 |
| 13 | 6.32 | 8.88 | 5.21 | 7.68 |
| 14 | 51.37 | 47.55 | 58.93 | 54.21 |
| 15 | 38.44 | 35.89 | 33.17 | 43.53 |
| 16 | 46.83 | 57.32 | 42.49 | 61.43 |
| 17 | 7.62 | 4.8 | 9.32 | 6.22 |
| 18 | 22.63 | 24.55 | 31.74 | 28.94 |
| 19 | 32.17 | 25.43 | 29.54 | 39.42 |
| 20 | 15.32 | 17.44 | 12.43 | 19.9 |

**Table 7 .** **Impact of long-term storage at -80˚C:** **the reading of anti-PLA2R antibodies in clear serum samples for 7 months**

| Patients | control | sealed | unsealed |
| --- | --- | --- | --- |
| 1 | 26.36 | 22.37 | 31.91 |
| 2 | 53.72 | 49.37 | 68.41 |
| 3 | 23.57 | 32.08 | 38.05 |
| 4 | 163.82 | 155.64 | 195.92 |
| 5 | 88.37 | 28.63 | 30.57 |
| 6 | 471.94 | 460.37 | 501.33 |
| 7 | 277.39 | 268.59 | 290.42 |
| 8 | 20.5 | 30.48 | 37.4 |
| 9 | 38.41 | 41.49 | 52.85 |
| 10 | 84.28 | 77.46 | 44.26 |
| 11 | 79.36 | 72.59 | 89.52 |
| 12 | 21.27 | 28.15 | 30.03 |
| 13 | 15.58 | 8.62 | 25.72 |
| 14 | 155.82 | 140.42 | 188.42 |
| 15 | 16.14 | 21.27 | 26.24 |
| 16 | 31.3 | 29.08 | 30.03 |
| 17 | 195.38 | 183.73 | 221.44 |
| 18 | 28.63 | 23.55 | 36.46 |
| 19 | 31.23 | 38.41 | 43.37 |
| 20 | 388.28 | 363.48 | 421.11 |
| 21 | 14.89 | 11.7 | 18.76 |
| 22 | 23.63 | 21.95 | 31.78 |

**Table 8 . Impact of long-term storage at -80˚C: the reading of anti-PLA2R antibodies in clear serum samples for 12 months**

| Patients | control | sealed |
| --- | --- | --- |
| 1 | 32.41 | 28.52 |
| 2 | 322.41 | 309.55 |
| 3 | 19.11 | 18.57 |
| 4 | 506.61 | 444.75 |
| 5 | 229.75 | 258.07 |
| 6 | 32.5 | 39.6 |
| 7 | 19.17 | 22.14 |
| 8 | 177.39 | 195.74 |
| 9 | 88.42 | 92.48 |
| 10 | 42.43 | 47.11 |
| 11 | 24.14 | 30.37 |
| 12 | 73.29 | 75.3 |
| 13 | 19.15 | 28.1 |
| 14 | 210.42 | 235.21 |
| 15 | 441.39 | 490.95 |
| 16 | 66.35 | 72.43 |
| 17 | 22.76 | 28.44 |
| 18 | 52.98 | 61 |
| 19 | 54.21 | 61.94 |
| 20 | 164.29 | 155.73 |
| 21 | 130.32 | 139.52 |

**Table 9 .** **Impact of physical and mechanical disturbance.**

| Patients | control | 1 hour | 8 hours |
| --- | --- | --- | --- |
| 1 | 2.38 | 3.01 | 3.11 |
| 2 | 5.06 | 26.62 | 29.77 |
| 3 | 200.52 | 221.93 | 187.68 |
| 4 | 18.8 | 20.46 | 20.83 |
| 5 | 55.37 | 69.85 | 66.5 |
| 6 | 90.99 | 81.49 | 84.2 |
| 7 | 8.64 | 10.13 | 11.9 |
| 8 | 60.55 | 67.72 | 65.63 |
| 9 | 157.58 | 145.83 | 140.95 |
| 10 | 5.21 | 5.13 | 5.03 |
| 11 | 30.57 | 32.32 | 37.3 |
| 12 | 104.26 | 101.46 | 105.84 |
| 13 | 12.74 | 15.45 | 15.56 |
| 14 | 4.19 | 3.51 | 3.71 |
| 15 | 134.37 | 103.2 | 119.91 |
| 16 | 15.35 | 18.42 | 17.33 |
| 17 | 427.22 | 472.67 | 491.88 |
| 18 | 198.48 | 204.28 | 195.29 |
| 19 | 95.38 | 91.77 | 108.35 |
| 20 | 59.32 | 65.37 | 68.94 |
| 21 | 107.94 | 118.95 | 123.3 |

**Table 10 . Impact of storage at -4˚C:** **The reading of anti-PLA2R antibodies in clear serum samples.**

| Patients | 0 week | 1 week | 3 weeks | 6 weeks |
| --- | --- | --- | --- | --- |
| 1 | 42.38 | 35.92 | 49.21 | 65.35 |
| 2 | 5.06 | 6.16 | 4.37 | 6.54 |
| 3 | 47.4 | 56.51 | 62.55 | 81.87 |
| 4 | 18.8 | 18.51 | 22.81 | 33.55 |
| 5 | 55.37 | 47.53 | 69.14 | 78.09 |
| 6 | 51.65 | 68.84 | 54.99 | 83.23 |
| 7 | 8.64 | 12.35 | 7.93 | 13.71 |
| 8 | 194.31 | 200.52 | 219.114 | 269.84 |
| 9 | 145.17 | 157.93 | 195.86 | 254.09 |
| 10 | 117.08 | 105.21 | 156.16 | 195.37 |
| 11 | 30.57 | 30.24 | 28.75 | 47.23 |
| 12 | 80.35 | 85.32 | 97.26 | 104.67 |
| 13 | 12.74 | 14.1 | 11.04 | 17.44 |
| 14 | 84.19 | 75.03 | 81.28 | 92.06 |
| 15 | 134.37 | 138.94 | 124.84 | 139.88 |
| 16 | 88.47 | 72.53 | 96.36 | 98.47 |
| 17 | 170.39 | 177.48 | 170.22 | 185.35 |
| 18 | 32.77 | 35.67 | 31.84 | 40.72 |
| 19 | 83.76 | 88.49 | 75.69 | 101.47 |
| 20 | 58.29 | 55.81 | 65.61 | 61.47 |
| 21 | 64.38 | 60.53 | 69.41 | 77.38 |

**Table 11 . Impact of storage at -4˚C: The reading of anti-PLA2R antibodies in turbid serum samples.**

| Patients | 0 week | 1 week | 3 weeks | 6 weeks |
| --- | --- | --- | --- | --- |
| 1 | 29.83 | 31.13 | 40 | 52.36 |
| 2 | 111.6 | 108.45 | 118.4 | 144.9 |
| 3 | 21.28 | 21.8 | 22.21 | 29.11 |
| 4 | 55.73 | 49.92 | 52.73 | 71.49 |
| 5 | 82.54 | 89.64 | 97.69 | 105.74 |
| 6 | 68.34 | 85.43 | 72.43 | 95.39 |
| 7 | 18.2 | 17.62 | 18.16 | 21.49 |
| 8 | 150.73 | 144.62 | 169.58 | 175.92 |
| 9 | 133.86 | 139.88 | 153.65 | 164.99 |
| 10 | 11.9 | 11.13 | 9.24 | 15.96 |
| 11 | 126.98 | 120.64 | 123.68 | 141.94 |
| 12 | 47.33 | 52.02 | 58.08 | 63.22 |
| 13 | 11.65 | 11.85 | 9.05 | 16.54 |
| 14 | 98.44 | 101.84 | 115.83 | 129.94 |
| 15 | 186.48 | 180.62 | 197.49 | 208.93 |
| 16 | 22.47 | 24.19 | 21.23 | 55.15 |
| 17 | 42.36 | 55.41 | 61.84 | 79.78 |
| 18 | 79.57 | 72.44 | 86.47 | 98.56 |
| 19 | 68.48 | 61.84 | 67.43 | 89.45 |
| 20 | 97.47 | 106.68 | 96.76 | 120.96 |

**Table 12 . Impact of storage at -4˚C: The reading of anti-PLA2R antibodies in haemolytic serum samples.**

| Patients | 0 week | 1 week | 3 weeks | 6 weeks |
| --- | --- | --- | --- | --- |
| 1 | 3.81 | 3.03 | 3.29 | 2.62 |
| 2 | 629.64 | 625.79 | 612.43 | 714.3 |
| 3 | 667.43 | 639.14 | 709.27 | 787.58 |
| 4 | 65.23 | 63.17 | 71.64 | 94.66 |
| 5 | 14.79 | 12.72 | 13.46 | 19.22 |
| 6 | 300.68 | 340.89 | 353.23 | 404.39 |
| 7 | 264.07 | 248.48 | 246.31 | 265.24 |
| 8 | 98.46 | 95.7 | 105.39 | 110.4 |
| 9 | 101.55 | 95.46 | 110.5 | 118.53 |
| 10 | 34.58 | 38.77 | 39.15 | 53.23 |
| 11 | 8.47 | 11.8 | 9.53 | 12.58 |
| 12 | 33.9 | 35.76 | 40.6 | 39.53 |
| 13 | 85.2 | 79.48 | 93.56 | 95.3 |
| 14 | 452.88 | 420.63 | 469.5 | 489.53 |
| 15 | 287.9 | 274.38 | 298.43 | 311.37 |
| 16 | 197 | 171.3 | 226.15 | 235.16 |
| 17 | 167.33 | 165.46 | 155.99 | 188.57 |
| 18 | 55.58 | 58.34 | 67.48 | 79.4 |
| 19 | 79.3 | 72.47 | 88.42 | 95.38 |
| 20 | 194.89 | 180.42 | 190.48 | 239.87 |

**Table 13 . Impact of storage at -4˚C: The reading of anti-GBM antibodies in clear serum samples.**

| Patients | 0 week | 1 week | 3 weeks | 6 weeks |
| --- | --- | --- | --- | --- |
| 1 | 39.61 | 43.68 | 68.38 | 52.37 |
| 2 | 55.85 | 51.84 | 63.94 | 71.53 |
| 3 | 86.94 | 79.42 | 93.54 | 95.22 |
| 4 | 5.75 | 3.4 | 4.27 | 4.37 |
| 5 | 7.4 | 8.49 | 6.83 | 6.48 |
| 6 | 111.75 | 118.41 | 124.64 | 139.79 |
| 7 | 77.46 | 79.48 | 76.97 | 85.31 |
| 8 | 5.98 | 3.59 | 4.38 | 7.67 |
| 9 | 19.21 | 17.21 | 20.29 | 23.23 |
| 10 | 2.76 | 5.09 | 2.62 | 3.89 |
| 11 | 42.88 | 36.95 | 39.52 | 64.21 |
| 12 | 39.74 | 45.79 | 37.31 | 39.53 |
| 13 | 33.8 | 38.25 | 44.51 | 59.68 |
| 14 | 3.77 | 2.06 | 5.17 | 4.55 |
| 15 | 64.89 | 50.52 | 69.46 | 76.84 |
| 16 | 74.93 | 71.58 | 74.23 | 93.24 |
| 17 | 9.79 | 10.31 | 8.84 | 7.46 |
| 18 | 21.46 | 19.8 | 17.29 | 31.06 |
| 19 | 69.53 | 74.24 | 79.41 | 88.53 |
| 20 | 82.99 | 88.63 | 95.32 | 113.62 |
| 21 | 63.74 | 69.42 | 75.26 | 86.42 |

**Table 14 .** **Impact of storage at -4˚C: The reading of anti-MPO antibodies in clear serum samples.**

| Patients | 0 week | 1 week | 3 weeks | 6 weeks |
| --- | --- | --- | --- | --- |
| 1 | 39.61 | 43.68 | 48.38 | 62.37 |
| 2 | 8.79 | 10.31 | 12.84 | 9.46 |
| 3 | 40.74 | 43.57 | 57.42 | 65.7 |
| 4 | 52.87 | 48.53 | 45.22 | 41.27 |
| 5 | 7.4 | 8.49 | 11.83 | 17.48 |
| 6 | 33.8 | 38.25 | 44.51 | 59.68 |
| 7 | 28.53 | 23.54 | 22.78 | 21.45 |
| 8 | 85.37 | 79.42 | 75.32 | 91.33 |
| 9 | 19.21 | 17.21 | 25.29 | 18.23 |
| 10 | 2.76 | 5.09 | 4.62 | 3.89 |
| 11 | 74.75 | 79.54 | 83.2 | 93.54 |
| 12 | 21.46 | 19.8 | 47.29 | 71.06 |
| 13 | 5.75 | 3.4 | 9.27 | 8.37 |
| 14 | 15.3 | 13.66 | 11.23 | 19.46 |
| 15 | 19.53 | 23.54 | 17.3 | 16.33 |
| 16 | 2.08 | 4.17 | 3.02 | 2.01 |
| 17 | 5.98 | 3.59 | 7.38 | 7.67 |
| 18 | 63.91 | 59.43 | 57.1 | 71.32 |
| 19 | 14.97 | 12.31 | 11.3 | 12.18 |
| 20 | 39.55 | 35.23 | 41.67 | 49.52 |

**Table 15 . Impact of storage at -4˚C: The reading of anti-PR3 antibodies in clear serum samples.**

| Patients | 0 week | 1 week | 3 weeks | 6 weeks |
| --- | --- | --- | --- | --- |
| 1 | 136.83 | 147.24 | 176.63 | 195.48 |
| 2 | 13.45 | 12.74 | 14.87 | 25.64 |
| 3 | 18.42 | 16.31 | 22.41 | 21.48 |
| 4 | 22.68 | 28.41 | 21.73 | 38.95 |
| 5 | 12.83 | 16.75 | 14.4 | 18.97 |
| 6 | 118.43 | 127.24 | 112.79 | 139.08 |
| 7 | 67.33 | 59.24 | 71.42 | 95.3 |
| 8 | 42.75 | 49.32 | 57.31 | 71.57 |
| 9 | 4.26 | 4.96 | 8.27 | 7.76 |
| 10 | 94.32 | 101.21 | 87.37 | 127.75 |
| 11 | 5.87 | 4.72 | 5.65 | 11 |
| 12 | 59.64 | 52.41 | 65.36 | 79.45 |
| 13 | 49.52 | 42.36 | 53.84 | 61.44 |
| 14 | 4.03 | 3.26 | 3.18 | 3.51 |
| 15 | 104.05 | 93.85 | 103.48 | 122.89 |
| 16 | 38.65 | 33.51 | 35.66 | 43.65 |
| 17 | 125.3 | 113.85 | 138.5 | 145.73 |
| 18 | 7.14 | 7.97 | 8.79 | 11.22 |
| 19 | 3.44 | 3.15 | 5.38 | 4.85 |
| 20 | 113.23 | 118.42 | 138.51 | 152.76 |

**Table 16 . Impact of storage at room temperature: The reading of anti-PLA2R antibodies in clear serum samples.**

| Patients | 0 day | 1 day | 7 days |
| --- | --- | --- | --- |
| 1 | 139.42 | 130.68 | 159.44 |
| 2 | 6.89 | 7.5 | 8.24 |
| 3 | 42.22 | 41.84 | 58.71 |
| 4 | 27.18 | 40.13 | 38.53 |
| 5 | 13.04 | 16.46 | 11.19 |
| 6 | 72.73 | 74.41 | 79.68 |
| 7 | 26.33 | 28.76 | 30.01 |
| 8 | 44.62 | 38.18 | 41.25 |
| 9 | 120.9 | 132.42 | 127.75 |
| 10 | 156.18 | 161.61 | 167.27 |
| 11 | 92.57 | 84.41 | 87.47 |
| 12 | 24.38 | 20 | 19.49 |
| 13 | 32.06 | 32.34 | 36.3 |
| 14 | 147.56 | 152.53 | 197.56 |
| 15 | 88.77 | 93.83 | 64.43 |
| 16 | 47.95 | 56.84 | 50.95 |
| 17 | 173.52 | 172.81 | 190.11 |
| 18 | 48.1 | 43.34 | 59.67 |
| 19 | 73.38 | 62.24 | 95.22 |
| 20 | 95.01 | 81.25 | 123.86 |

**Table 17 . Impact of storage at room temperature: The reading of anti-PLA2R antibodies in turbid serum samples.**

| Patients | 0 day | 1 day | 7 days |
| --- | --- | --- | --- |
| 1 | 3.17 | 3.01 | 5.89 |
| 2 | 511.47 | 455.98 | 608.16 |
| 3 | 455.98 | 498.33 | 601.79 |
| 4 | 94.66 | 98.47 | 108.94 |
| 5 | 2.35 | 2.1 | 2.95 |
| 6 | 396.74 | 426.58 | 452.89 |
| 7 | 126.31 | 104.32 | 169.7 |
| 8 | 24.53 | 21.42 | 23.62 |
| 9 | 155 | 164.32 | 188.57 |
| 10 | 4.72 | 3.02 | 8.77 |
| 11 | 19.42 | 16.34 | 22.84 |
| 12 | 37.31 | 31.53 | 46.96 |
| 13 | 264.07 | 270.33 | 310.64 |
| 14 | 202.99 | 199.54 | 286.15 |
| 15 | 48.61 | 41.8 | 45.83 |
| 16 | 79.84 | 64.21 | 83.52 |
| 17 | 99.5 | 108.45 | 147.9 |
| 18 | 325.62 | 311.42 | 398.65 |
| 19 | 200.4 | 231.42 | 310.5 |
| 20 | 84.24 | 72.31 | 103.86 |

**Table 18 . Impact of storage at room temperature: The reading of anti-PLA2R antibodies in haemolytic serum samples.**

| Patients | 0 day | 1 day | 7 days |
| --- | --- | --- | --- |
| 1 | 5.41 | 8.73 | 11.03 |
| 2 | 95.24 | 103.24 | 140.96 |
| 3 | 113.52 | 102.32 | 148.54 |
| 4 | 74.21 | 84.21 | 98.65 |
| 5 | 56.61 | 63.22 | 67.33 |
| 6 | 12.64 | 10.72 | 22.94 |
| 7 | 42.62 | 53.78 | 64.56 |
| 8 | 68.31 | 59.53 | 79.43 |
| 9 | 20.54 | 22.25 | 24.19 |
| 10 | 37.51 | 43.53 | 41.3 |
| 11 | 11.9 | 12.34 | 12.26 |
| 12 | 64.33 | 82.35 | 109.33 |
| 13 | 88.43 | 81.52 | 101.32 |
| 14 | 75.21 | 84.62 | 93.64 |
| 15 | 8.21 | 6.3 | 14.75 |
| 16 | 26.07 | 29.11 | 35.63 |
| 17 | 108.42 | 122.97 | 135.63 |
| 18 | 68.34 | 65.57 | 88.37 |
| 19 | 18.84 | 17.31 | 21.9 |
| 20 | 99.43 | 73.11 | 109.3 |
| 21 | 60.25 | 67.88 | 79.78 |

**Table 19 . Impact of storage at room temperature: The reading of anti-GBM antibodies in clear serum samples.**

| Patients | 0 day | 1 day | 7 days |
| --- | --- | --- | --- |
| 1 | 160.37 | 174.57 | 199.8 |
| 2 | 44.68 | 39.62 | 69.57 |
| 3 | 4.72 | 6.34 | 5.49 |
| 4 | 33.01 | 33.7 | 46.97 |
| 5 | 29.53 | 24.32 | 38.55 |
| 6 | 58.36 | 64.23 | 83.77 |
| 7 | 17.02 | 16.97 | 20.23 |
| 8 | 25.15 | 21.29 | 29.09 |
| 9 | 129.53 | 103.22 | 153.78 |
| 10 | 136.21 | 148.62 | 169.74 |
| 11 | 5.35 | 6.52 | 4.27 |
| 12 | 5.71 | 5.84 | 6.21 |
| 13 | 142.78 | 135.42 | 183.27 |
| 14 | 43.8 | 39.92 | 69.43 |
| 15 | 5.73 | 5.15 | 7.89 |
| 16 | 9.04 | 10.35 | 13.67 |
| 17 | 18.94 | 15.63 | 22.75 |
| 18 | 15.79 | 15.96 | 16.6 |
| 19 | 11.46 | 13.6 | 22.38 |
| 20 | 85.72 | 77.42 | 101.32 |
| 21 | 8.62 | 11.96 | 10.47 |

**Table 20 . Impact of storage at room temperature: The reading of anti-MPO antibodies in clear serum samples.**

| Patients | 0 day | 1 day | 7 days |
| --- | --- | --- | --- |
| 1 | 69.67 | 77.58 | 81.09 |
| 2 | 101.4 | 95.72 | 88.89 |
| 3 | 40.43 | 50.85 | 53.15 |
| 4 | 33.42 | 31.74 | 49.2 |
| 5 | 86.87 | 91.41 | 80.43 |
| 6 | 12.93 | 15.04 | 19.53 |
| 7 | 75.83 | 62.37 | 89.45 |
| 8 | 89.72 | 81.32 | 97.32 |
| 9 | 14 | 14.53 | 14.25 |
| 10 | 31.8 | 42.26 | 43.95 |
| 11 | 74.42 | 79.32 | 85.98 |
| 12 | 19.73 | 15.34 | 15.74 |
| 13 | 14.82 | 17.95 | 18.29 |
| 14 | 28.24 | 36.45 | 46.48 |
| 15 | 52.49 | 47.93 | 61.83 |
| 16 | 15.32 | 19.43 | 21.4 |
| 17 | 6.14 | 8.84 | 7.34 |
| 18 | 97.06 | 93.28 | 102.02 |
| 19 | 3.21 | 2.09 | 5.38 |
| 20 | 64.97 | 59.72 | 89.6 |

**Table 21 . Impact of storage at room temperature: The reading of anti-PR3 antibodies in clear serum samples.**

| Patients | 0 day | 1 day | 7 days |
| --- | --- | --- | --- |
| 1 | 136.83 | 159.66 | 185.45 |
| 2 | 14.63 | 12.44 | 13.64 |
| 3 | 8.87 | 9.18 | 13.22 |
| 4 | 73.5 | 69.43 | 98.6 |
| 5 | 53.87 | 49.76 | 45.93 |
| 6 | 2.79 | 2.16 | 4.84 |
| 7 | 3.5 | 3.26 | 2.03 |
| 8 | 39.76 | 33.62 | 46.74 |
| 9 | 22.97 | 28.64 | 39.71 |
| 10 | 5.27 | 5.63 | 4.13 |
| 11 | 5.55 | 6.18 | 11 |
| 12 | 47.92 | 45.83 | 37.54 |
| 13 | 79.54 | 85.37 | 101.73 |
| 14 | 55.37 | 52.84 | 72.76 |
| 15 | 11 | 12.83 | 10.6 |
| 16 | 110.64 | 119.55 | 138.95 |
| 17 | 68.94 | 63.11 | 87.04 |
| 18 | 22.78 | 25.83 | 34.11 |
| 19 | 18.54 | 18.98 | 15.36 |
| 20 | 32.73 | 39.62 | 30.2 |
